# Approaches to STI Prevention and Control in a Highly Decentralized State: An Integrative Review

**DOI:** 10.3389/frph.2022.725646

**Published:** 2022-07-06

**Authors:** Javier Gómez Castellá, Asunción Díaz Franco, Rosa Polo Rodríguez, Julia del Amo Valero

**Affiliations:** ^1^National Plan on AIDS, Ministry of Health, Madrid, Spain; ^2^University of Alcalá de Henares, Madrid, Spain; ^3^National Centre for Epidemiology, Institute of Health Carlos III (ISCIII), Madrid, Spain

**Keywords:** HIV, STI, Health policy, Strategic Plan, Spain

## Abstract

The Spanish health system is highly decentralized and Autonomous Regions (AR) are responsible for managing and implementing the National Strategic Plan for Prevention and Control of HIV and other Sexually Transmitted Infections (STI) (2013–2020) via autonomous plans adapted to the characteristics of each region. The aim of this study is to report on actions taken to specifically address STI prevention and control in the autonomous plans within the mainframe of the National Strategic Plan. An integrative review was performed to analyse the health plans and HIV/STI plans of the Spanish AR during the period of validity of the current National Strategic Plan; 2013–2020. Plans were selected attending to specificity and whether strategies were in force during the year 2020. Our gatherings show that plans are largely focused to HIV prevention and control and, to a lesser extent, to STI prevention. The analysis on pre-existing resources for care of STI varied by region, and epidemiological surveillance systems for STI notification were not completely adopted by all of them. Particularly noteworthy are information campaigns, pre- and post-exposure prophylaxis for HIV, and prevention activities in community settings. The remarkable lack of studies concerning STI resources and investment in Spain highlights the necessity of consensus-based tools for evaluation and future planning of STI prevention and control measures. The high degree of heterogeneity among plans for prevention of HIV infection and STIs also points out a high number of different areas for improvement in the development of new AR plans in HIV/STI.

## Introduction

Since 2000, and especially since 2010, the incidence of sexually transmitted infections (STI) has increased gradually to become a major public health problem throughout the world (1–4). In 2016, the World Health Organization (WHO) published its Global Health Sector Strategy on Sexually Transmitted Infections 2016–2021 (5), to achieve the objectives of the 2030 Agenda for Sustainable Development. The publication of this document was followed by STI strategies several countries, although these have been unevenly developed in the European Union.

In Spain, the National Strategic Plan for Prevention and Control of HIV and other STI (2013–2020) (“Strategic Plan”), is structured along national and international coordination, improvement of information systems, measures for the prevention of HIV and other STI, and diagnosis and treatment (6, 7). The current focus on STI is insufficient and should be boosted so it can address these infections in an integrated approach, yet differently from HIV infection. To do so, we must analyze the current situation of the strategic plans promoted by the Spanish government.

Autonomous Regions (AR) of Spain, under the coordination of the Ministry of Health, are responsible for management and planning of health care within their specific area of the country, including the implementation of the Strategic Plan adapted to the specific characteristics of each AR (8). However, there is a lack of information regarding the scope of the actions taken against HIV and STI in the different AR, as there are no studies in Spain of this kind.

The aim of the present article is to describe the actions taken against STI in the plans of the AR of Spain, as well as the degree of alignment with the strategic lines and objectives of the National Strategic Plan.

## Methods

An integrative review of the literature was performed to analyse the various health care plans and HIV/STI plans in force in the 17 AR and 2 Autonomous Cities in Spain during the period of validity of the current National Strategic Plan; 2013–2020 (9–11). The integrative review was performed systematically, according to the following steps: identification of the area of study; definition of the inclusion criteria; search for documentation; selection of relevant studies; design of a data collection card for each AR; interpretation of the results; and structured data synthesis.

The inclusion criteria were restricted to Spain and priority was given to searches for information in Spanish, although documents in co-official languages were also included. To be included, the documents had to fulfill at least 1 of the following criteria: plan for prevention and control of STI and/or HIV/AIDS; health plans; sexual health strategies; evaluation reports from autonomous health care plans or clinical protocols for management of STI.

Information was taken mainly from available sources on the websites of the Directorates General of the AR and the Ministry of Health. The Center for Documentation of Pedagogical Resources in HIV and AIDS (CDPR) of the non-profit organization SIDA STUDI was used to complement the information search, as was Google. We contacted the persons in charge of implementing STI plans in the AR in order to collect additional information. We combined key search terms including the name of each AR using the Boolean operator “AND”, as follows: “STI plan”, “HIV plan”, “Health plan”, “Health strategy”. All elements included in the descriptor “Health policy” were analyzed through the CDPR. The first search revealed 47 documents, 31 health plans and 16 health plan evaluation reports.

Studies were selected based on 2 criteria: specificity of the autonomous plan and whether the health care strategies were in force during the year 2020. In the case of a specific HIV/STI plan was not in force, we included the global health care plan of the AR if it was in force. In the case of AR that did not have a specific HIV/STI plan, we included global health plans and other relevant plans in force. The most recent evaluation reports were also included. During the second round of revision, we selected a total of 33 documents (24 health plans and 9 health care plan evaluation reports).

The evaluation of the measures in the autonomous plans was structured based on the 4 strategic lines and target population was evaluated according 3 axes of intervention brought together in the National Strategic Plan (6, 7). Data were collected systematically using a data collection form.

## Results and Discussion

We selected 24 autonomous plans corresponding to 17 AR. One AR did not have an autonomous plan, 8 had a specific HIV/STI plan, including an additional one from an Autonomous City, and the remainder had non-specific plans. Table 1 summarizes the list of documents included, showing whether the various health care strategies remain in force and the target populations for the plans.

**Table 1:** Summary of the plans from the various Autonomous Regions: specificity, duration, availability of evaluation reports, and target population.

**Autonomous Region**	**Title of the plan and year**	**Type of strategic plan**	**Anti-STI plans in force/not in force in 2020**	**Evaluation reports**	**Priority target populations^†^**
Galicia	Galician plan for HIV/AIDS and other sexually transmitted infections. 2015–2018 (12)	HIV/STI	In force	*Evaluationreportoftheanti*−*HIVandAIDSplaninGalicia*(2000−−2013)^‡^	People who live with HIV MSM PEP PID Migrants PPI Young people Women Health professionals General population
	Galician plan for HIV/AIDS and other sexually transmitted infections. Extension 2019–2022 (13)	HIV/STI			
Asturias	Program for prevention and for care of persons living with HIV/AIDS in Asturias: strategies for reducing the health care and social impact of HIV/AIDS in Asturias 2003–2007 (14)	HIV	Not in force	Evaluation of the program for prevention and care of people affected by HIV-AIDS in Asturias 2003–2017 (15)	Young people PEP PPI
Cantabria	Cantabria health plan 2014–2019 (16)	Health Plan	Not in force	Not available	General population Young people PID MSM Migrants
Basque Country	Strategic plan for prevention and control of HIV and other STIs, 2015–2018 (17)	HIV/STI	Not in force	AIDS and Sexually Transmitted Diseases Plan. 2018 Report (18)	General population MSM Young people PEP Migrants People who live with HIV
Navarre	Navarre health plan 2014–2020 (19)	Health Plan	In force	Not available	General population Young people
	Public health plan 2016–2020 (20)	Health Plan			
La Rioja	III Health plan for La Rioja 2015–2019 (21)	Health Plan	Not in force	Preliminary Evaluation Report of the II Health Care Plan of La Rioja. Situation as at December 31, 2013^‡^	General population
Aragón	Aragon health plan 2030 (22)	Health Plan	In force	Not available	General population
Cataluña	Plan for action against HIV and other STIs 2016–2020 (23)	HIV/STI	In force	Not available	General population Young people PID PEP MSM PPI
Community of Valencia	IV Plan de Salud de la Comunitat Valenciana 2016–2020 (24)	Health Plan	In force	Intermediate Evaluation Report 2018 of the IV Health Care Plan (25)	General population Young people PID
	Sexual and reproductive health strategy of the Community of Valencia 2017–2021 (26)	Health Plan			
Balearic Islands	HIV/AIDS strategy for the Balearic Islands (27)	HIV/STI	In force	Report on HIV/AIDS of the Balearic Islands 2018 (28)	General population Migrants Women Young people PID MSM PEP PPI Health care personnel
Murcia	Health plan 2010–2015 of the Region of Murcia (29)	Health Plan	Not in force	Evaluation of the health plan 2010–2015. Final report on evaluation of objectives (30)	General population
Castille-Leon	IV Health plan for Castille-Leon. Outlook 2020 (31)	Health Plan	In force	Evaluation report of the III health plan of Castille-Leon (2008–2012) (32)	General population
Community of Madrid	Healthy district strategy 2016–2019 (33)	Health Plan	In force	Not available	General population Young people PEP Trans persons
	Action plan for Health Madrid 2020 (34)	Health Plan			
Extremadura	Action plan for HIV/AIDS in Extremadura 2012–2015 (35)	HIV/STI	Not in force	Not available	General population Young people MSM PEP PID Migrants People who live with HIV Health care personnel
	Action plan for HIV and other STIs in Extremadura 2018–2021 (36)	HIV/STI	In force		
Castille - La Mancha	Health plan for Castille–La Mancha 2019–2025 (37)	Health Plan	Not in force	Not available	General population Young people MSM PEP PPI Health professionals
	Strategic plan for the prevention and control of HIV infection and other STIs 2014–2017 (38)	HIV/STI			
Andalusia	Andalusian plan for HIV/AIDS and other STIs (2010–2015): Extension 2016–2020 (39)	HIV/STI	In force	Not available	General population Young people MSM PEP PID PPI Migrants
Canary Islands	Health plan for the Canary Islands 2016–2017 (40)	Health Plan	Not in force	Evaluation report. Health Plan of the Canary Islands 2016–2017 (41)	General population Young people
Ceuta	II Action plan for HIV and other sexually transmitted infections 2015–2017 (42)	HIV/STI	Not in force	Not available	General population Young people MSM PEP PID PPI Migrants People who live with HIV Health workers

† *Legend of table: MSM, men who have sex with men; PEP, persons who engage in prostitution; PPI, persons in penitentiary institutions; PID, persons who inject drugs*.

‡ *Included in the health care plan, not as independent documents*.

The measures reported in the autonomous plans for HIV and other STI vary widely, with major differences depending on the region. Measures for STI are less developed than those for HIV infection. While this heterogeneity can be explained in part by regional differences in the AR, the poorer alignment with the National Strategic Plan in some areas is due to structural factors, probably associated with health care planning in each region, underinvestment in health care resources, and the prioritization or not of STI as a public health problem (19, 21, 22, 31). The review is structured by themed areas depending on the elements evaluated:

### Rationale, Relevance, and Situation Analysis

A section was established to review the rationale for drawing up different plans, with differences in exhaustiveness. The HIV/STI plans define their rationale and indication more clearly and are based mainly on the epidemiological evidence available when the plan was drawn up; however, the plans were more fully developed in the case of HIV than the other STI.

The epidemiological situation analysis was performed in all plans, although more detail was supplied HIV/STI plans. HIV and syphilis epidemiological baseline analysis is common to all AR plans, whereas for the other STI it varies widely according to the information systems in the different AR. Some AR have developed additional epidemiological surveillance systems to assess the sexual and reproductive health of the target population, as well as the degree of knowledge about HIV and STI (12–14, 23, 39).

Several factors are considered accountable for variations in the analysis of the epidemiologic situation in the various plans; the year of publication, the characteristics of the surveillance systems in the AR, and the establishment of specific notification systems, as well as the inclusion of new notifiable diseases in the notification system from the year 2015 onward (2). Surveillance systems should be adapted to limit biases of under-reporting of STI, and reliable, recent data should be made available. A major aspect of the epidemiological analysis is that it was only completed before the implementation of a health or HIV/STI plan. In the different evaluation reports consulted, we found epidemiological data and reports, although the analysis of the epidemiological situation in order to evaluate the different strategic lines and actions envisaged was not carried out in all of them (25, 28, 32, 41). When this analysis was performed, epidemiological trends in these AR show that actions undertaken to control STI within the development of the strategic plans have not been effective, which is also reflected in epidemiological data for STI at national level (4, 18, 30).

With respect to the analysis of pre-existing resources, in the case of non–HIV-specific plans, the analysis of health care resources was wide-reaching and did not include a specific analysis of resources earmarked for care of patients with STI. Similarly, in the case of HIV/STI–specific plans, there was no detailed situation analysis of the resources allocated for care of STI. Only 2 out of all the AR plans included an exhaustive situation analysis of the health care resources assigned for treatment of STIs in Spain (26, 39).

Analysis of the situation and of the capacity of the health system is a necessary step in the development of a strategic plan aimed at the prevention and control of HIV infection and other STI (43). The characterization of health resources and centers for care of patients with STI and current epidemiological surveillance systems represents a first key step when designing specific plans for HIV/STI. However, this first step is often omitted in the drafting of AR plans or is not examined in depth in the situation analysis. Despite its importance, there is a remarkable lack of studies concerning STI resources and investment in Spain, thus making further planning on STI prevention and control less efficient. These studies highlight the importance of STI centers and diagnostic resources characterization as an essential area for improvement in the AR with respect to prevention and control of HIV and STI.

### Coordination of the Response to HIV/STI and Epidemiological Surveillance

In general terms, various coordination actions have been established with the Ministry of Health and the Secretariat of the National AIDS Plan, between different AR, or both. Although the implementation of mechanisms for coordination with the Ministry of Health is an objective of the National Strategic Plan, the establishment of such mechanisms is not made explicit in several AR plans, although there are many modalities of cooperation between the central government and the AR. Regarding healthcare planning and development of strategic plans, consensus-based tools should be developed between the different AR and central government in order to systematize the evaluation process and apply it to health care plans in different areas, not exclusively HIV and STI.

With respect to coordination with the community sector, the construction of cooperative networks with NGO or collaboration with community bodies in the area of prevention of HIV/STI and health education varies by region. Collaborative actions are not explicitly stated in one third of the AR plans reviewed, although *ad hoc* collaborations have been established in specific areas with respect to HIV and STI prevention between the community sector and the ARs governments.

All of the AR plans include the commitment to maintaining the current epidemiological surveillance systems. As for improvements in epidemiological surveillance systems in the AR and the inclusion of new notifiable diseases STI (*Chlamydia trachomatis* and *Lymphogranuloma venereum*) in the year 2015 (44), only 10 AR envisage including these diseases as objectives in their plans. The inclusion of new notifiable infections is still being implemented in several AR. Particularly interesting is the creation of AR surveillance systems that complement those already in place with information regarding sexual behaviors, community testing or wider STI notification systems (12–14, 23, 36, 39).

Another important area for improvement within epidemiologic surveillance systems is the reporting of the results of rapid diagnostic tests in the community setting. Although the HIV self-test has been available in Spain for use outside hospitals since 2017 (45), there are no surveillance systems at state or at AR level to collect the epidemiological information from community care centers to enable the strategic plans to be adapted to the needs of the target population. Initiatives have been undertaken within the framework of European projects for improving notification systems in the community setting (46). These systems, however, should be implemented at national level and cover all AR to be representative of the real situation of early diagnosis in community settings. The Ministry of Health has developed a specific platform for reporting results of rapid HIV tests in community settings (REDCO-VIH) which will permit to determine the situation of HIV diagnosis in community care centers, an essential step for developing more specific actions in HIV/STI prevention.

Cooperation between public administrations and the community to develop preventive HIV and other STIs actions and policies is essential, as reported in various international organisms (47, 48). Nevertheless, leadership in prevention policies falls to government bodies, which should be responsible and accountable for coordinating the preventive actions. Within the framework of HIV and other STI prevention, the measures reported by each AR are well aligned with the objectives set out in the Strategic Plan, although specific regional differences can still be found. Of particular interest is the variation between the AR in terms of regulation of rapid diagnostic testing in community setting. Performance of rapid STI tests in community setting is not specifically regulated at state level, with each region individually establishing the necessary conditions (45). A recent systematic review concerning HIV testing outside of healthcare facility settings shows that despite community testing poses a significant advantage in diagnosing HIV infection (and other STI), particularly among people at high risk of infection with difficult access to healthcare settings, there are still many legal barriers in the majority of European countries regarding delivery of community-based testing by non-medical staff and use of home-sampling or self-testing kits (49).

### Health Promotion and Prevention of HIV Infection and STI

Health education activities and preparation of information campaigns on HIV infection and STI prevention are common to all plans, though more developed in the specific HIV and STI plans, with greater emphasis on HIV than on other STI. The number and period of the training activities and campaigns vary between AR, although World AIDS Day is a common milestone. Training activities on prevention of HIV infection and STI are also common to all the AR plans and target both the general population and various groups of particular epidemiological interest depending on the characteristics of each AR.

With respect to activities aimed at primary prevention of HIV infection and other STI, educational interventions on risk groups are common to all plans. These interventions focus on promoting condoms use and safe sex and harm reduction programs in specific groups, especially in persons who inject drugs. There are also various interventions by the community sector in groups at high risk of acquiring HIV and other STI. Another important element within the framework of primary prevention is the drafting and implementation of protocols for post-exposure prophylaxis (PEP) and pre-exposure prophylaxis (PrEP) to prevent HIV infection, with differences by AR. Also common to all is the promotion and funding of proven effective preventive activities through calls for NGO funding.

As for early diagnosis, considerable differences were observed between HIV infection alone and HIV infection and other STI. Various models for the early detection of HIV infection have been put in place in the AR, as set out in the relevant plans. These include rapid HIV testing in primary care and community settings and, albeit less developed, promotion of HIV self-tests sold in pharmacies. Early diagnosis of other STI varies between the autonomous plans. In some cases, rapid testing for diagnosis of STI (syphilis) has been implemented, although in most AR plans, there are no details on which specific type of diagnostic testing is performed as part of actions aimed at boosting early diagnosis of STI. No references to early diagnosis of HIV or STI were found in the plans of 5 AR (19, 20, 29, 31, 40). Protocols targeting the study of contacts in the case of STI were only explicitly developed as part of the objectives in the plans of 3 ACs (12, 13, 21, 23).

### Improvement of Health Outcomes

Only 7 AR envisage specific measures to improve management of STI (14, 16, 17, 23, 24, 26, 33, 34, 39). Of note, specific STI care protocols have been created, existing resources have been reinforced with emphasis on primary care in collaboration with other units, and clinical history taking has been improved to include sexual lifestyles. The plans of the remaining AR promote maintenance and enhancement of services aimed at HIV infection, with no specific emphasis on management of STI (12, 13, 27, 35–38).

As for measures to guarantee the continuity of care for patients with STIs and HIV infection, the only explicit measures were those aimed at the establishment of specific care circuits for the follow-up of persons with STI in 7 ARs (14, 16, 23, 24, 26, 33, 34, 37–39). The main ones were designation of reference centers and professionals for follow-up, mainly of HIV infection and—albeit to a lesser extent—of STI, in addition to specific referral protocols in the case of STI in order to ensure follow-up between care levels. Particularly noteworthy is the coordination between primary care services and follow-up and analysis of STI contacts in specialist care centers, with marked emphasis on the follow-up of patients at primary care.

### Stigmatization, Discrimination, and Access to the Health System

As for the measures in place to fight against stigmatization and discrimination, the main actions envisaged in the various plans are aimed at contesting false myths and beliefs with respect to HIV (with almost no actions for other STI), actions against stigmatization and discrimination, and information campaigns to raise awareness among the general public. Cooperation with local government and NGO to carry out actions to reduce stigmatization in people with HIV is common to all autonomous plans. Implementation of these measures varies somewhat.

In the case of access to the health system—understood as factors that hinder or facilitate contact with or benefits of health care or other resources—the measures set out in the plans are aimed mainly at people with HIV (23, 24, 26, 35–39, 42). Very few measures aim at improving access for persons with non-HIV STI.

Lastly, while measures have been taken to address the stigma associated with HIV infection in Spain (50), there is still much room for improvement in this area, and other STI should also be considered a source of stigmatization and subsequent discrimination. In addition, measures should be developed to improve access to the health system for people who acquire an STI.

### Study Limitations

This review has several limitations. Firstly, health plans can differ from real life when addressing measures taken to control HIV and STI. Secondly, it has not been possible to develop an appropriate indicator set to measure the implementation and results of the different actions in the strategic plans. This paper shows the enormous heterogeneity of plans and the lack of evaluation reports, highlighting several areas of improvement in this field.

## Conclusions

Despite its importance in the development of new strategic plans regarding STI prevention and control, there is a remarkable lack of studies concerning STI resources and investment in Spain, thus making further planning on STI prevention and control less efficient. Development of consensus-based tools for evaluation and future planning of HIV and STI strategies is utterly necessary as a way to highlight strengths and weaknesses and provide proper HIV/STI prevention and control impact measures. There is also a high degree of heterogeneity among plans for prevention of HIV and STI with room for specific actions, particularly in the case of STI. The development of specific STI plans should be promoted. There are numerous areas for improvement in the development of new HIV/STI plans, especially in terms of performing a situation analysis before implementation and carrying out specific actions to address the care of patients with STI, as well as stigmatization and discrimination.

## Author Contributions

JG conducted the review and wrote the manuscript. RP and JA supervised the project. All authors commented on and edited earlier versions and read and approved the final manuscript.

## Conflict of Interest

The authors declare that the research was conducted in the absence of any commercial or financial relationships that could be construed as a potential conflict of interest.

## Publisher's Note

All claims expressed in this article are solely those of the authors and do not necessarily represent those of their affiliated organizations, or those of the publisher, the editors and the reviewers. Any product that may be evaluated in this article, or claim that may be made by its manufacturer, is not guaranteed or endorsed by the publisher.
